# Buscogeny: A BUSCO leveraged phylogenomic tree builder

**DOI:** 10.1007/s10123-025-00752-6

**Published:** 2026-01-23

**Authors:** John Webster, Toni A. Chapman

**Affiliations:** NSW Department of Primary Industries and Regional Development, Sydney, NSW Australia

**Keywords:** BUSCO, Phylogenomic, Fungal, Taxonomy, Phylogenetic, Automated, Bioinformatic, Pipeline, Recombination filtering

## Abstract

Accurately resolving evolutionary relationships among organisms is a cornerstone of evolutionary biology, with phylogenetic trees serving as critical tools for this purpose. While single-gene phylogenies are widely used, they often fail to capture the full complexity of evolutionary processes, particularly in organisms with complex or variable genomes. Concatenated gene alignments, built from multiple orthologous genes, offer a more robust framework but are challenging to generate, especially in taxa with incomplete or poorly annotated genomes such as fungi. To address this challenge, we present Buscogeny (Busco Phylogeny), an open-source command-line tool that automates the construction of concatenated gene alignments and phylogenetic trees using BUSCO-derived single-copy orthologs. Buscogeny integrates key features including genome quality assessment, ortholog extraction, multiple sequence alignment, gap filtering, and recombination filtering, followed by automated phylogenetic inference. By allowing users to select ortholog datasets of varying resolutions and tailor filtering thresholds, Buscogeny is adaptable to both closely related and more distantly related taxa. We demonstrate the utility of Buscogeny in phylogenetic analyses of both bacterial and fungal genomes, including complex groups such as *Alternaria*, where annotation and genome completeness vary significantly. The streamlined, reproducible workflow provided by Buscogeny makes BUSCO-based phylogenetic inference accessible to researchers across disciplines, enabling robust evolutionary insights from whole-genome data.

**Supplementary Information:**

The online version contains supplementary material available at 10.1007/s10123-025-00752-6.

## Introduction

Understanding the evolutionary relationships among organisms is fundamental to the field of evolutionary biology. Phylogenetic trees, depicting the evolutionary history of species, are essential tools for elucidating these relationships. Traditionally, these trees were constructed based on individual gene sequences, leading to the creation of single gene trees.

However, it is increasingly recognised that single gene trees may not always accurately represent the complex evolutionary history of organisms, especially when genes undergo different evolutionary processes or when gene duplication and loss events occur (Haggerty 2009). To address these challenges, researchers have turned to concatenated gene alignments, a process in which multiple genes are concatenated and subsequently aligned into a single supermatrix (Sanderson 2003).

Concatenated gene trees have gained prominence due to their ability to provide a more comprehensive and reliable representation of evolutionary relationships (Crawford and Snitkin 2021; Dettman and Eggertson 2021). By incorporating information from multiple genes, concatenated trees can mitigate the effects of gene-specific evolutionary events, offering a more robust framework for phylogenetic analysis.

Developing sets of genes within a specific lineage on which a concatenated gene alignment may be generated can be challenging. A common approach for bacteria might be to find an MLST scheme and perform an in-silico search for the alleles within a scheme, through methods such as BLAST, extracting gene coordinates or searching annotation files (Jolley et al. 2018). However, this approach is limited in resolution for schemes utilising only a small number of loci, and not suitable for organisms without a defined scheme.

Fungal phylogenies present unique challenges due to the complexities of fungal genomes, their high variability, and often incomplete genomic annotations. Fungi are a highly diverse group of organisms with both ecological and economic significance, ranging from pathogens to symbionts (Adnan 2022). Their phylogenetic relationships, however, have been difficult to resolve using traditional approaches, primarily due to the sparsity of suitable genomic data (Dettman and Eggertson 2021). Many fungal species lack well-annotated genomes, and gene markers are often difficult to identify across distantly related taxa. The availability of draft fungal genome sequences has increased over the past decade, but high-quality, annotated genomes remain relatively scarce, especially for non-model species.

Other core gene phylogenetic methods, such as generating a core gene alignment from pan-genome studies, such as Panaroo (Tonkin-Hill 2020), can generate very large alignments of hundreds of genes. However, this requires prior annotation of sequences and the tools generally only work for bacterial genomes. The process of fungal genome annotation is not so trivial and fungal genome phylogenetics could benefit from similar concatenation methods (Foissac 2008; Mohanta and Al-Harrasi 2021).

BUSCO (Benchmarking Universal Single-Copy Orthologs) is a widely used tool designed to assess the completeness of genome assemblies by identifying a set of highly conserved single copy orthologs (Simão 2015). These genes are expected to occur as single copies in nearly all species within a given lineage, reflecting essential cellular functions that are maintained through evolution. Because they are both ubiquitous and evolutionarily stable, the presence, absence, or duplication of these orthologs provides a reliable measure of assembly completeness and potential redundancy. BUSCO uses pre-computed ortholog datasets derived from OrthoDB and employs sequence similarity searches and hidden Markov models to identify orthologs in a genome, transcriptome, or protein set. Ortholog databases are available to download manually or through BUSCO across Eukaryotes, Bacteria and Viruses. These orthologs, prevalent across evolutionary lineages, serve as essential markers of genome integrity, as a full suite of complete orthologs indicates the completeness of a genome assembly. In contrast, missing, fragmented or duplicated orthologs suggest that assemblies may be incomplete or contain erroneously assembled segments.

In an effort to automate a consistent process of deriving phylogenetic trees from large concatenated gene supermatrices, we present Buscogeny, a computational tool designed to streamline the process of constructing concatenated sequence alignments and phylogenetic trees from BUSCO derived orthologs. By harnessing the power of BUSCO and its databases, Buscogeny identifies reliable single copy orthologs, overcomes the challenges associated with fungal genome annotation, and facilitates the generation of concatenated gene alignments for accurate phylogenetic analyses from bacteria, viruses or eukaryotes.

In this study, we explore the effectiveness of Buscogeny in elucidating the evolutionary relationships of multiple taxa. Through this tool, we aim to provide a valuable resource for future phylogenetic studies in organisms with diverse and difficult to elucidate evolutionary histories.

## Materials and methods

### Buscogeny implementation

**Fig. 1:**
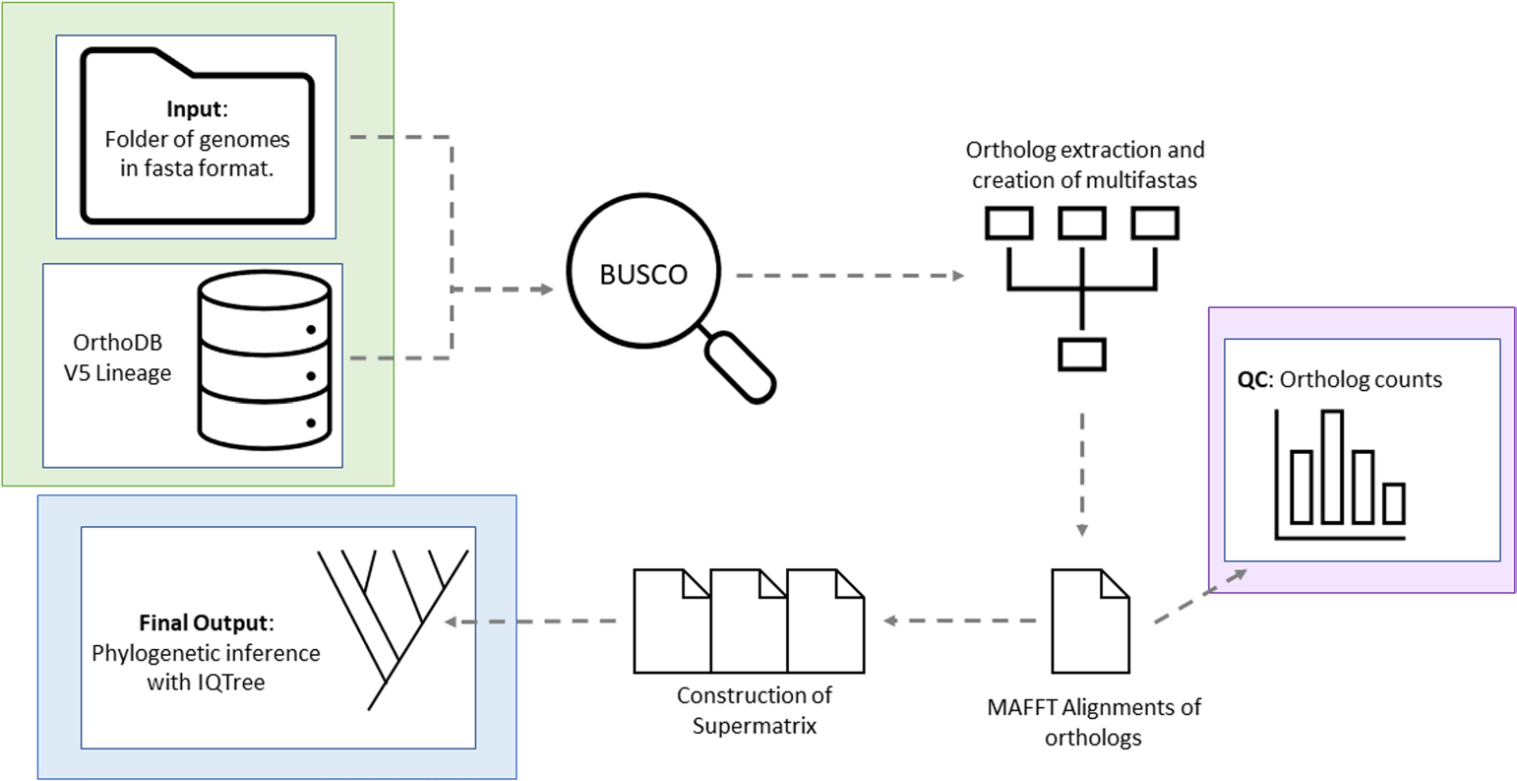
Buscogeny Workflow. Required input files (green box) include a folder of genomes in fasta format and an OrthoDB lineage database. All intermediary files, including a QC graph (purple box), are output in the Buscogeny output directory. The final output of the pipeline consists of an IQTree maximum likelihood tree (blue box)

Buscogeny is a specialised bioinformatics tool for constructing phylogenetic trees and is implemented in Python 3. Buscogeny leverages the BUSCO (Benchmarking Universal Single-Copy Orthologs) (Simão 2015) framework to identify conserved orthologs across a range of taxa levels with accompanying orthoDBs. This approach aids in creating robust and accurate phylogenetic trees based off conserved single copy orthologs. Buscogeny is accessible via GitHub athttps://github.com/Jwebster89/Buscogeny. A generalised workflow for Buscogeny is depicted in Fig. 1.

Buscogeny can be installed by cloning the git repository and using the package manager Conda to manage the installation of dependencies as outlined in the GitHub documentation. Buscogeny utilises Python 3 and modules for BioPython (Cock 2009), Pandas (McKinney 2011), Numpy (Oliphant 2006)and Alive bar (Almeida 2025), while running standard bioinformatic tools such as mafft (Katoh 2002), IQTree (Hoang 2018; Kalyaanamoorthy 2017; Minh 2020), clipkit (Steenwyk 2020), ClonalFrameML (Didelot and Wilson 2015), maskrc-svg (Kwong 2019)and BUSCO (Simão 2015). Documentation for installation and usage is provided in the associated Github Repository.

At time of publication, Buscogeny requires three inputs: the folder of genome assemblies (-i/--input), the location of the OrthoDB database (-d/--db), and an output prefix (-o/--output). In addition, a number of optional arguments allow users to customise their analyses. These include the number of threads (-t/--threads, default 8), the ClipKIT gappy threshold for alignment trimming (-g/--gappy_threshold, default 0.05), and the proportion of alignments an isolate may be missing from before exclusion (-e/--exclude_threshold). Users can also specify whether to align protein or nucleotide sequences (-s/--seq_type, default protein) and optionally enable recombination filtering (-r/--rc_filt). Standard help and version flags are also available (-h/--help). The most up to date parameters can be found on Buscogeny’s GitHub page.

### BUSCO

The BUSCO analysis forms a crucial component of Buscogeny, underpinning the initial phase of phylogenetic tree construction. This initial phase is dedicated to identifying single copy-orthologs in the provided genome set. Various taxonomic levels of orthoDB are available for download (https://busco-data.ezlab.org/v5/data/lineages/) and can be tailored to the organism of interest (Kriventseva 2019). This step is fundamental in determining the sequences to be used in the final supermatrix.

As orthoDB may be available at various taxonomic levels, the resolution of the phylogenetic output may be tailored depending on the organism, with lower level taxonomic orthoDBs containing a larger contingent of conserved sequences. For example, *Xylella fastidiosa* may be examined with the V5 databases at the Domain (bacteria_odb10, *n* = 124), Class (gammaproteobacterial_odb10, *n* = 366) or Order (xanthomonadales_odb10, *n* = 1,152) taxonomic levels.

**Fig. 2:**
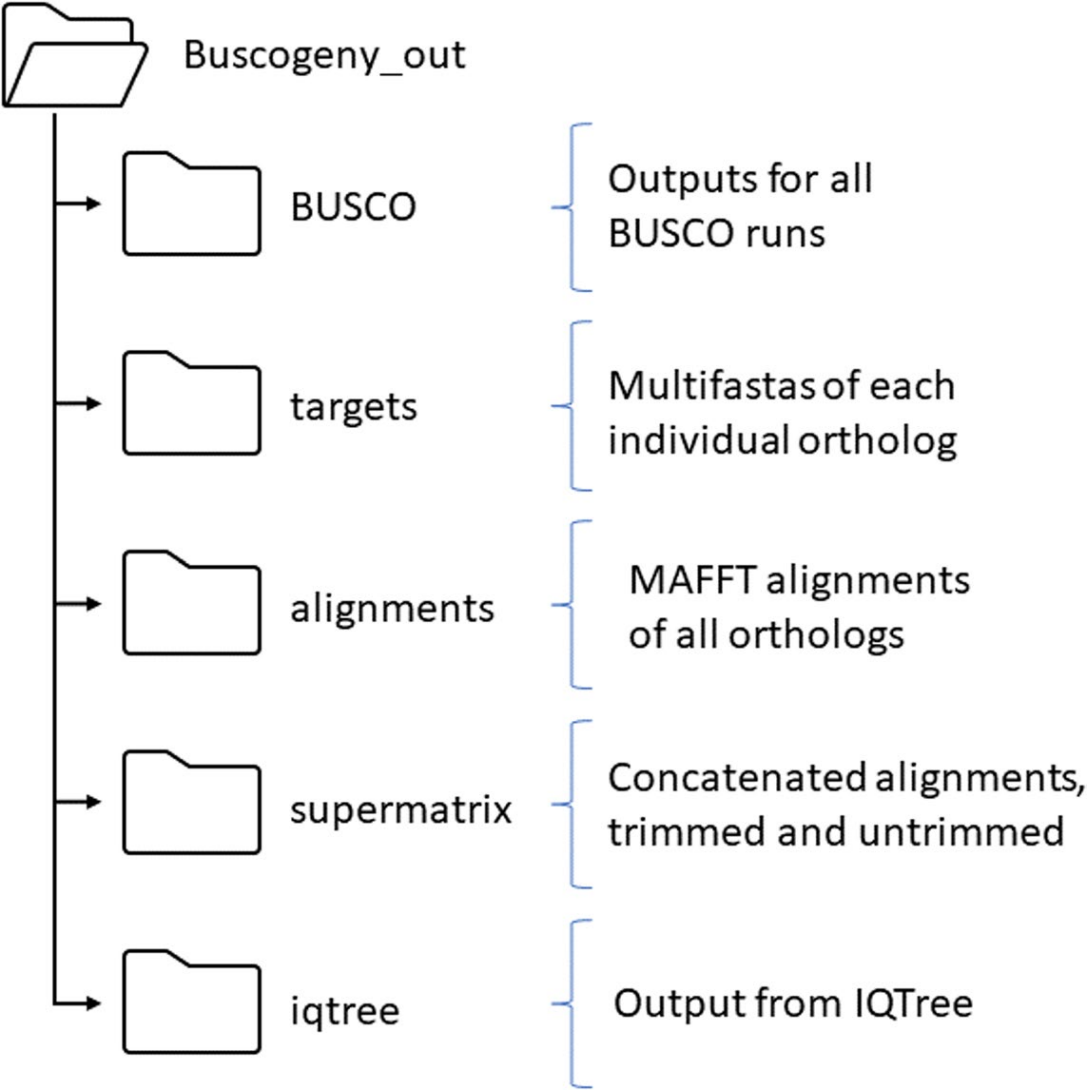
Buscogeny output folder structure. Folders include all intermediary output created by Buscogeny, including fasta files of target sequences, alignments, trimmed and untrimmed supermatrices, and IQTree phylgenetic inference

The same can also be performed for other domains, e.g. *Alternaria alternata* may be examined at the Domain (eukaryota_odb10), Phylum (fungi_odb10), Division (ascomycota_odb10) or Order (pleosporales_odb10). Upon execution, Buscogeny runs the ‘BUSCO’ Python method to invoke BUSCO as a subprocess call, using the provided (--db) orthoDB as the lineage dataset. Buscogeny checks the prefix supplied for -o (--output) and creates an output folder named “< prefix> _Buscogeny_out” if it is not found, to store all of its output (Fig. 2).

As it’s input, Buscogeny takes a folder of genomes, for which it runs BUSCO and all subsequent steps on each genome provided in the input directory. Each genome’s BUSCO results are maintained in a separate subdirectory named after the genome file, ensuring easy traceability and access. These results include ortholog sequences identified by BUSCO, which then form the basis for subsequent steps in the Buscogeny pipeline, where the sequences are further processed for alignment and phylogenetic tree construction.

As of BUSCO version 5.4.0, single copy orthologs are saved in protein fasta, nucleotide fasta and GFF files, allowing for the creation of concatenated phylogenies using gene or protein sequences with the -s (--seq_type) flag in Buscogeny.

### Target ortholog processing and alignment

Following the BUSCO processing of genomes, Buscogeny utilises two Python methods to retrieve the identified orthologs and prepare them for phylogenetic tree construction. A list of BUSCO targets is extracted from the provided orthoDB, and for each target ortholog, Buscogeny searches through the BUSCO output directories and extracts the corresponding ortholog sequences from each input genome. These sequences are then compiled into multifasta files, one for each target ortholog.

Once multifasta files are generated, the *target_alignments* method is employed to align each ortholog cluster of sequences. This method is crucial for ensuring that the ortholog sequences from each input genome are properly aligned, a prerequisite for accurate phylogenetic analysis. For each multifasta file, Buscogeny uses MAFFT to perform sequence alignment, automatically determining the input sequence direction for optimal alignment.

### Plotting ortholog counts

As an important quality metric, the assessment of ortholog counts across input genomes is performed. This analysis is facilitated by two key methods in the Python code. Each file, representing an alignment in FASTA format produced in the previous step, is parsed to extract the genome identifiers. Buscogeny counts the occurrences of each unique genome identifier, and stores these counts in a Python dictionary structure, producing an aggregated count of each genome. This count provides a quantitative measure of orthologs in each genome, which is then visualised using the matplotlib library in the form of a bar plot. This plot allows for the visual inspection as to the completeness of the supplied genomes.

Later stages of Bucogeny involve removing gap positions from the supermatrix before phylogenetic inference. In this context, the incompleteness of genomes, as indicated by lower ortholog counts, corresponds to a reduction in the final alignment length. Therefore, genomes with excessive missing information, as revealed by this analysis, might be considered for exclusion from further analysis.

### Supermatrix construction

Following alignment of individual orthologs, Buscogeny constructs a supermatrix, a concatenation of all ortholog alignments. Buscogeny implements an exclusion threshold to identify genomes missing from more than a specified proportion of alignments. By default, this threshold is set to 20% (customisable using the -e or --exclude_threshold flag) in order to remove poorly performing genomes. Users may change this to be more strict or flexible in the removal of genomes that fail to be present in a specified number of alignments.

Genomes falling above this threshold are flagged and subsequently removed from the final analysis. This step prevents the inclusion of genomes with substantial missing data that could otherwise lead to significant reductions in alignment length. The remaining alignments are then concatenated and written out to a clustal file using the BioPython library. In addition, further refinement of the supermatrix is performed by trimming gap positions with clipkit (https://jlsteenwyk.com/ClipKIT/) using the clipkit gappy mode and a default gappyness of 0.05 (customisable with -g, --gappy_threshold). The resulting trimmed clustal alignment is written to a file in the output directory.

### Phylogenetic inference

The final step in Buscogeny produces a phylogenetic tree from the trimmed ortholog supermatrix. IQ-Tree with default model testing is used to determine sequence type and substitution model, with Ultrafast bootstrap approximation utilising 1,000 replicates.

### Recombination filtering

If recombination filtering is activated using the --rc_filt flag the supermatrix (step 2.5) is constructed as an XMFA file with 1000 gap characters inserted between gene partitions. Recombination is inferred using ClonalFrameML (Didelot and Wilson, 2015 ), which incorporates the maximum likelihood phylogenetic tree from IQ-TREE and the XMFA alignment. Recombination is inferred using the expectation-maximisation (EM) algorithm (-em) to estimate recombination parameters and branch lengths, with 100 pseudo-bootstrap replicates (-emsim 100) used during parameter estimation. Recombination is masked from the concatenated gene alignment using maskrc-svg and the ClonalFrameML output and the masked alignment used as input to generate the final maximum-likelihood phylogeny using IQ-Tree.

## Results

### Phylogenomics of fungal genomes

Phylogenomics of fungal genomes presents unique challenges, particularly in the construction of multi-gene phylogenetic trees. To compare genes, first ab-initio gene prediction needs to be performed and often requires external evidence in the form of RNA and/or proteomic data. As such, fungal genome annotation is notably more complex when compared to bacteria, where genomes can be annotated swiftly using tools like Prokka and Bakta.

Annotating fungal genomes requires appropriate processing resources and bioinformatic know-how. One of the main aims of Buscogeny was to assist in the phylogenomic inference of fungal genomes using a methodology that was agnostic to prior annotation by the user. This method leverages the BUSCO framework to identify single-copy orthologs.

BUSCO employs a two-phased approach in its genome mode to identify single-copy orthologs. Initially, TBLASTN uses amino acid consensus sequences from a given database to identify genomic sequences that likely contain each BUSCO gene. This is followed by AUGUSTUS, which delineates precise gene structures on the regions identified from the TBLASTN results. HMMER then assigns scores to these sequences, facilitating a preliminary classification of their completeness. In the second phase, AUGUSTUS is retrained with parameters derived from initially identified complete genes to enhance gene prediction accuracy. Further searches with TBLASTN, retraining with AUGUSTUS and classification with HMMER help in refining the identification and classification of genes missing from the first phase of BUSCO.

By utilising these orthologs, Buscogeny constructs a concatenated supermatrix and performs phylogenetic inference from a large alignment without the need for external or user provided gene prediction and annotation. This approach simplifies the process of producing a phylogenetic tree inferred from whole genomes while also performing a thorough assessment of genome completeness and integrity.

**Fig. 3:**
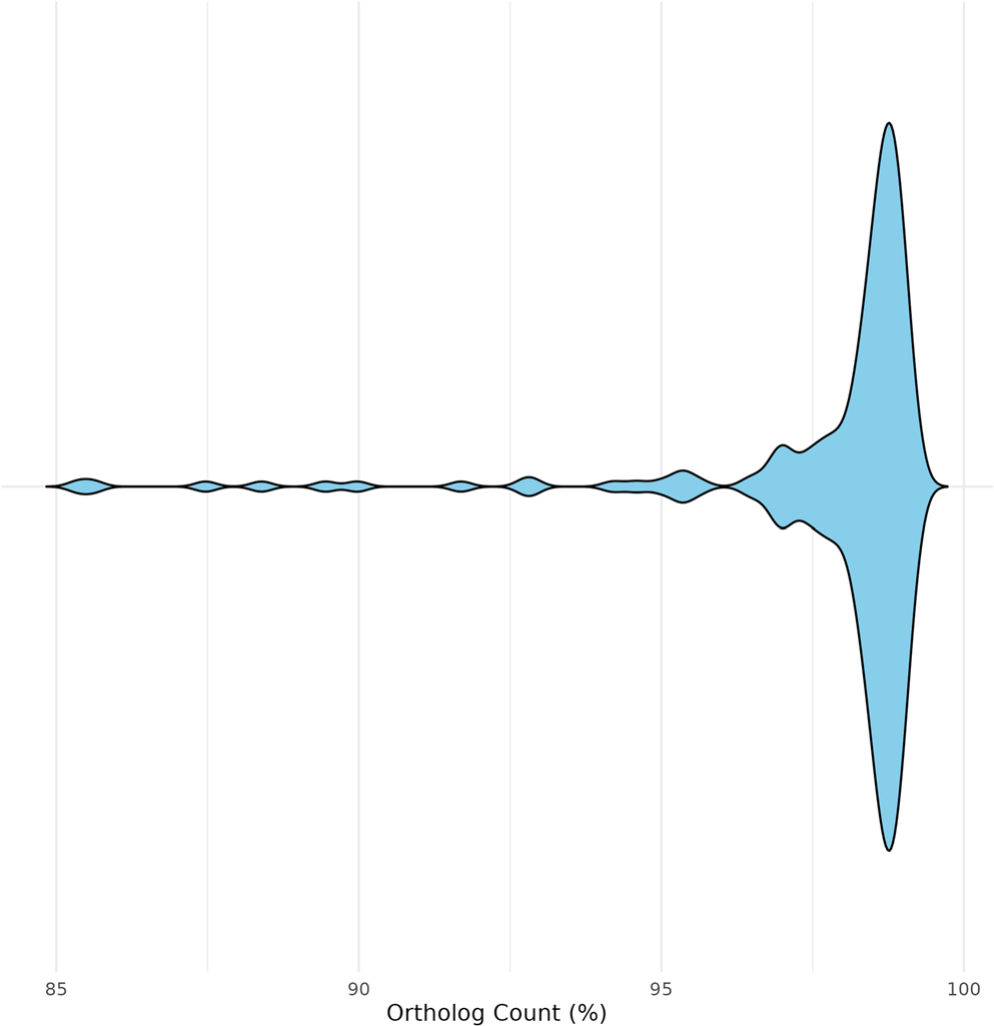
Total ortholog counts (fungi_odb10, *n* = 758) from each genome of *Alternaria* (*n* = 178) as assessed by BUSCO

As an example, all publicly available *Alternaria* genomes were downloaded with NCBI_genome_download (https://github.com/Jwebster89/NCBI_genome_download), and processed with Buscogeny using the fungi_odb10 database, the nucleotide alignment option, and a gappyness threshold of 0.05. Genome completeness (single copy orthologs) assessed by BUSCO was between 85.3% and 99.2% (Fig. 3). Isolates were filtered for genomes too distant or low genome completeness before the phylogenetic tree was inferred.

The taxonomy of *Alternaria* has undergone significant revisions over the years, with multiple genera being synonymised into *Alternaria*, leading to changes in species definitions and classifications. This has resulted in the current division of the genus into 26 sections. The largest, *Alternaria* section *Alternata*, initially comprised approximately 60 species but was later reduced to 11 based on phylogenetic analyses. More recent phylogenomic studies have further refined this to four major species clades: *A. alternata*, *A. longipes*, *A. arborescens*, and *A. gaisen*.

**Fig. 4:**
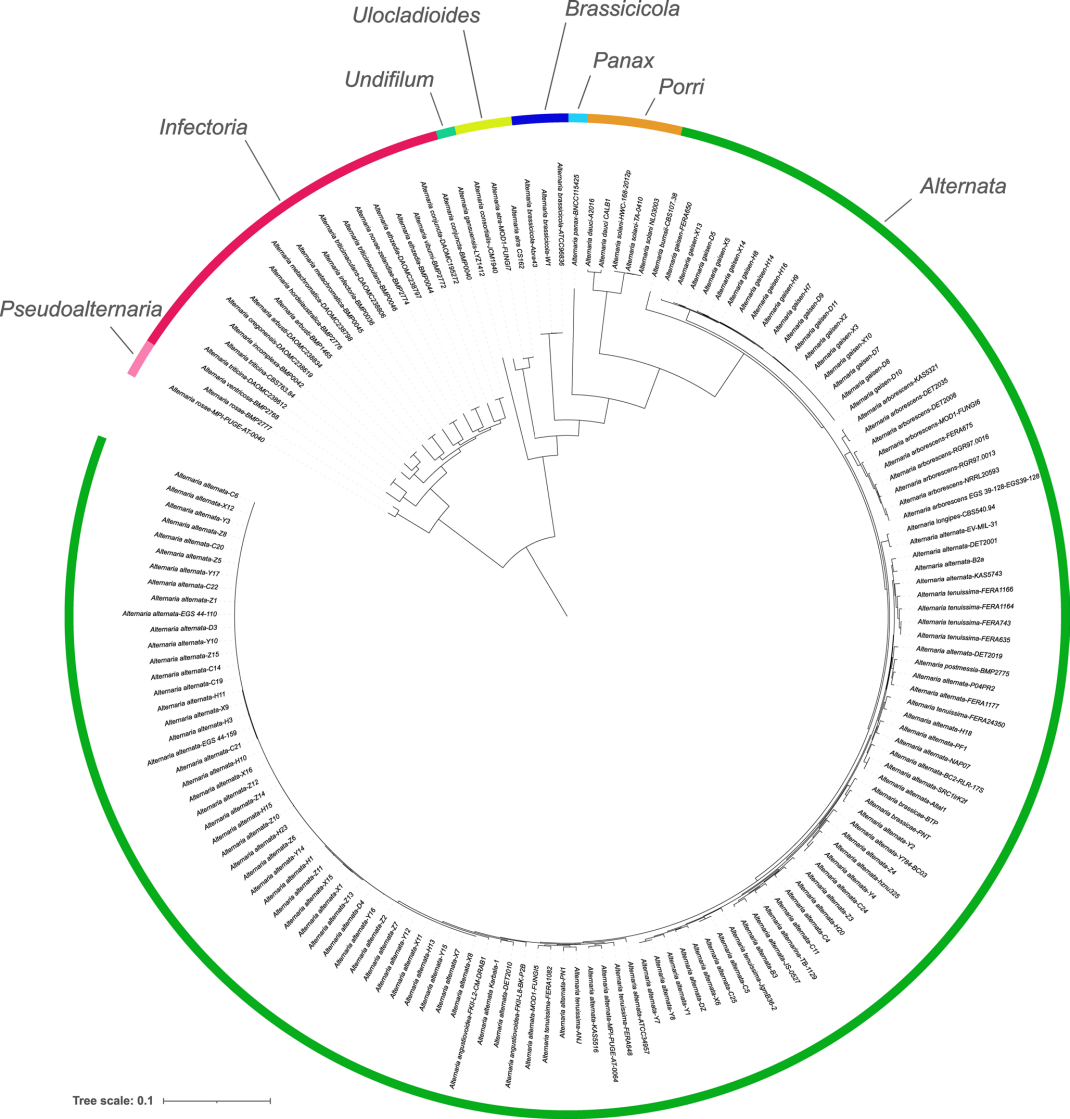
Maximum likelihood phylogeny of the genus *Alternaria* utilising the fungi_odb10 (*n* = 758 orthologs) in Buscogeny

BUSCO-based phylogenetic analyses of the *Alternaria* genus using available genomes revealed a strong congruence between major clades and the currently defined sections (Fig. 4). Although only one A. longipes genome was available, making it challenging to confirm the previously described A. longipes clade, the phylogenetic inference clearly delineated *A. arborescens*, *A. alternata*, and *A. gaisen*.

**Fig. 5:**
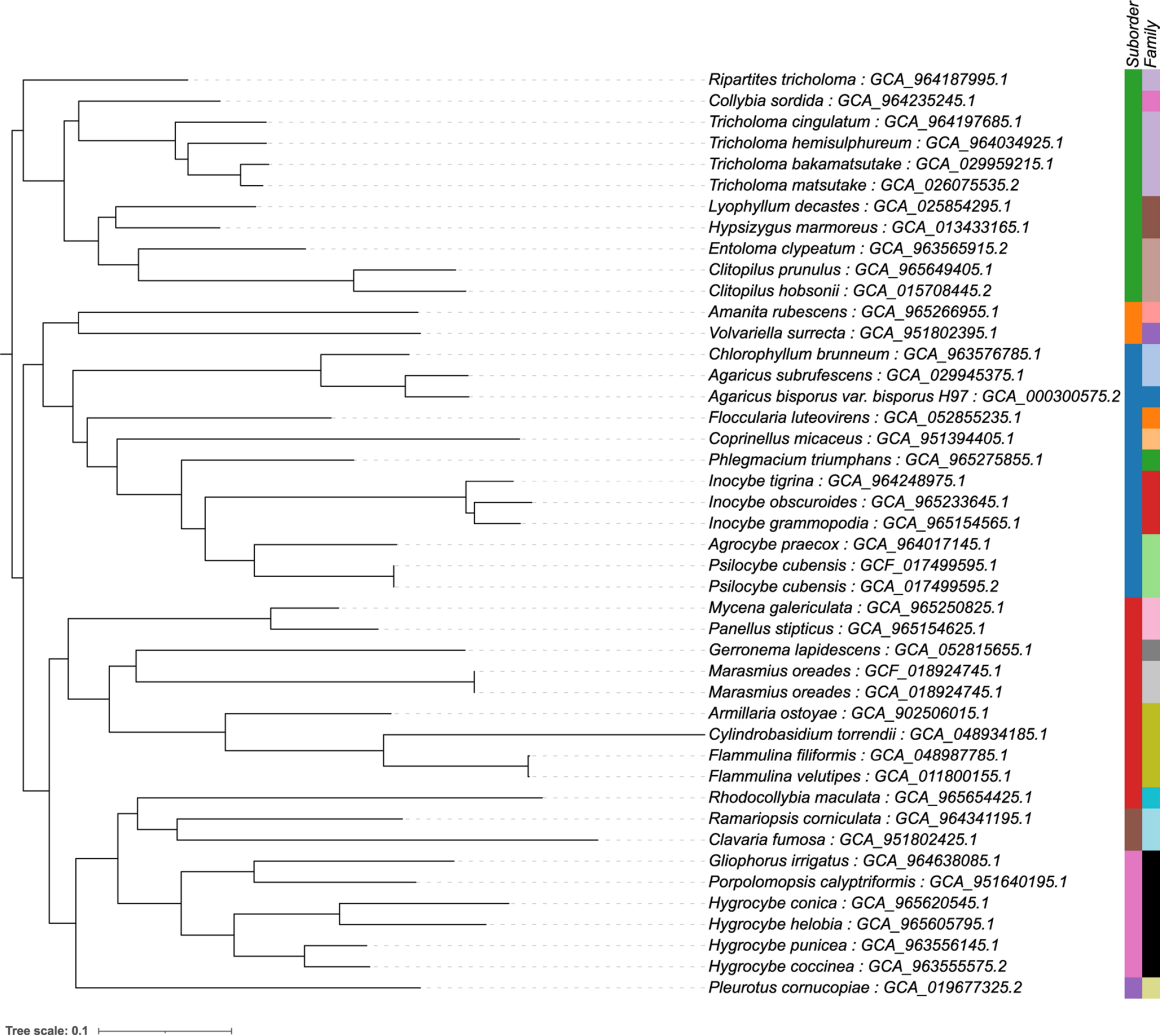
Maximum likelihood phylogeny of the order Agaricales utilising the basidiomycota_odb12 (*n* = 2409) in Buscogeny

To further evaluate Buscogeny’s applicability across diverse fungal lineages, a phylogenetic analysis was performed on genomes from the order Agaricales, representing the Basidiomycota. The Agaricales, or euagarics clade, is the largest group of mushroom-forming fungi, encompassing more than half of all known homobasidiomycete species (Hibbett 1997; Matheny 2006). Historically, classifications relied on morphological characteristics such as hymenophore type and spore colour (Fayod 1889; Fries 1874, 1821; Kuhner 1980; Singer 1949), but molecular studies have shown many of these groupings to be artificial, leading to the recognition of several well-supported clades within the order (Matheny 2006). Genomes from representative taxa were processed using the basidiomycota_odb10 lineage dataset with default Buscogeny parameters. The resulting maximum-likelihood phylogeny recovered the major suborders within Agaricales, including Tricholomatineae, Pluteineae, Agaricineae, Marasmiineae, Clavariineae, Hygrophorineae and Pleurotineae (Fig.5). These suborders correspond closely to those recognised in recent multilocus and phylogenomic analyses (Dentinger 2016; Vizzini 2024; Wang 2024). Although the precise ortholog set differs from those used in traditional multigene studies, Buscogeny effectively reproduced recognised higher-level relationships within the order, demonstrating its ability to generate robust genome-scale phylogenies for complex fungal lineages.

### Bacterial phylogenetic inference with buscogeny

**Fig. 6:**
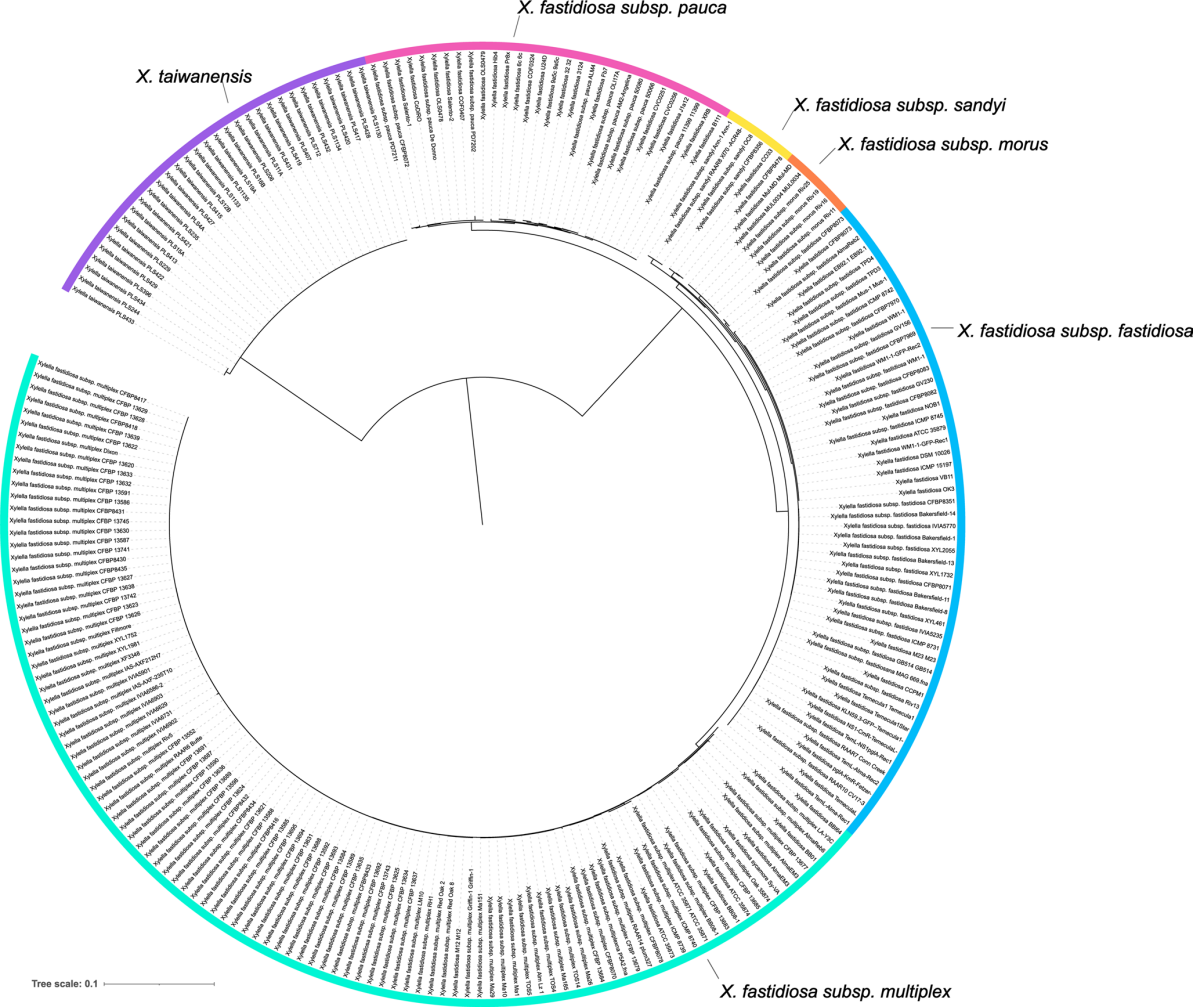
Maximum likelihood phylogeny of the genus Xyella utilising the bacteria_odb10 (*n* = 124 orthologs) in Buscogeny

The phylogenetic inference of the Genus *Xylella* was performed using Buscogeny with three orthoDB lineage sets, bacteria, xanthomonadales and gammaproteobacteria (Fig. 6).

The analysis included a set of 234 publicly available genomes from two different species within the Genus *Xylella*, notably *Xylella fastidiosa* and *Xylella taiwanensis*, including *X. fastidiosa* subspecies *pauca*, *morus*, *sandyi*, *fastidiosa* and *multiplex*.

Two principal clades corresponding to *Xylella fastidiosa* and *Xylella taiwanensis* were visible from the phylogenetic inference with Buscogeny. In addition, the *Xylella fastidiosa* clade had several well-defined sub-clades relating to subspecies of Xylella, including *X. fastidiosa* subsp. *fastidiosa*, *X*. *fastidiosa* subsp. *sandyi*, *X*. *fastidiosa* subsp. *morus*, and *X. fastidiosa* subsp. *multiplex*. Each subspecies forming a distinct grouping within the X. fastidiosa clade, indicating clear genetic delineations among them.

**Fig. 7:**
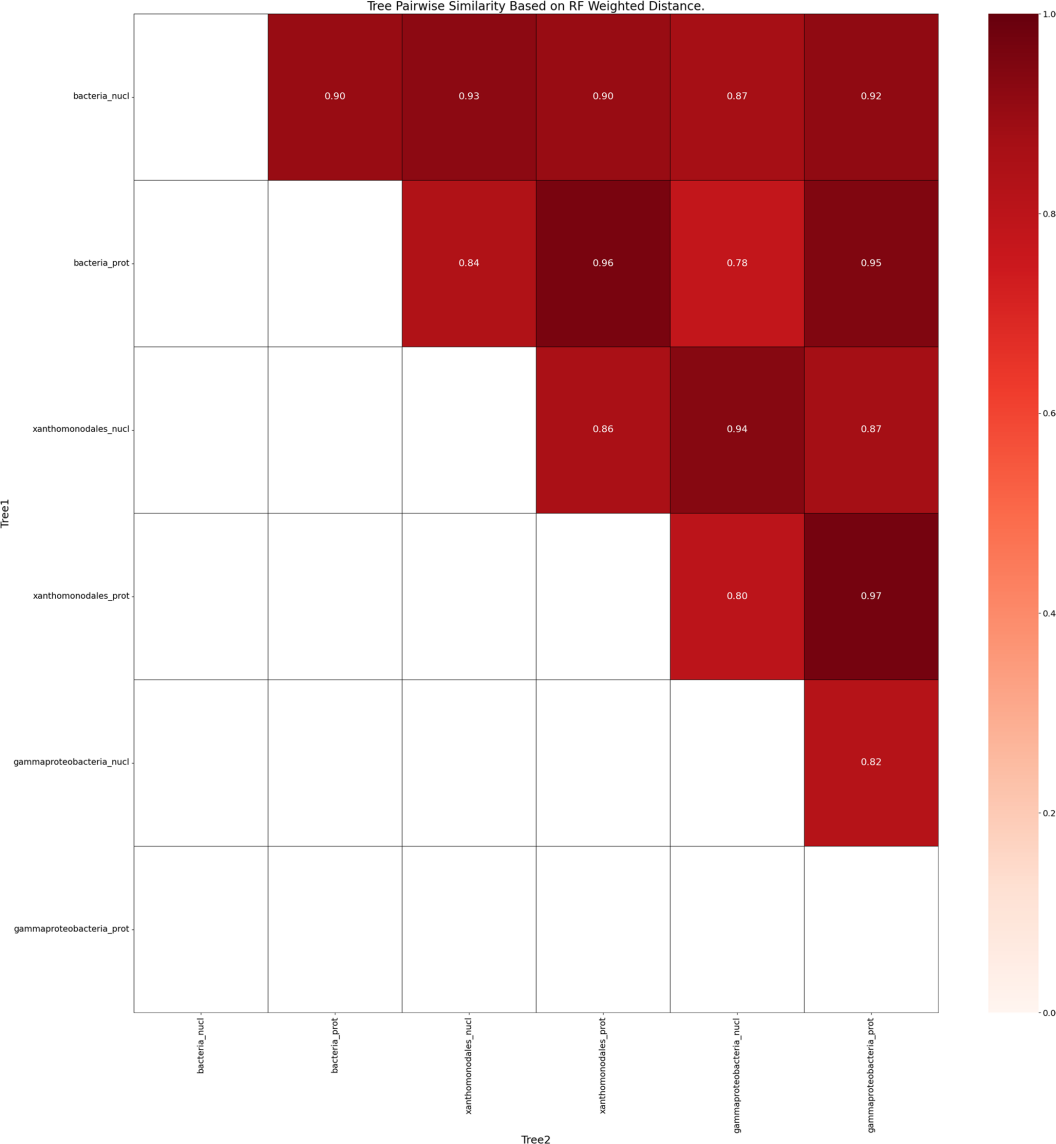
Upper triangular matrix of transformed Weighted Robinson-Foulds distances from three orthoDB lineage sets, comparing similarity of branching order between resulting Buscogeny trees tested with nucleotide and protein BUSCO options

Similarity metrics of nucleotide and protein alignments of *Xylella* genomes using the bacteria_odb10, gammaproteobacterial_odb10, and xanthomonadales_odb10 were computed using a weighted Robinson-Foulds distance measure (Fig. 7). All tree combinations had a high similarity to each other, suggesting a robust conservation of phylogenetic structure across different orthologous database classifications, and indicating a methodological consistency that effectively captures phylogenetic relationships irrespective of the database used.

### Viral phylogenetic inference with buscogeny

Phylogenetic inference of viral genomes presents a distinct set of challenges compared to fungi and bacteria. Viruses often encode compact genomes with fewer conserved genes, and phylogenies are typically built from a more limited set of core proteins. For example, baculoviruses (family *Baculoviridae*) are classified into four main genera (Alpha-, Beta-, Gamma- and Deltabaculovirus), with alphabaculoviruses further subdivided into groups I and II. Previous studies have shown that the current criterion for classification relies on distances and phylogenies derived from the 38 core baculovirus proteins, sometimes supplemented with the major occlusion body protein (MOBP) as a 39th core gene (Cerrudo 2023; Trentin 2019).

**Fig. 8:**
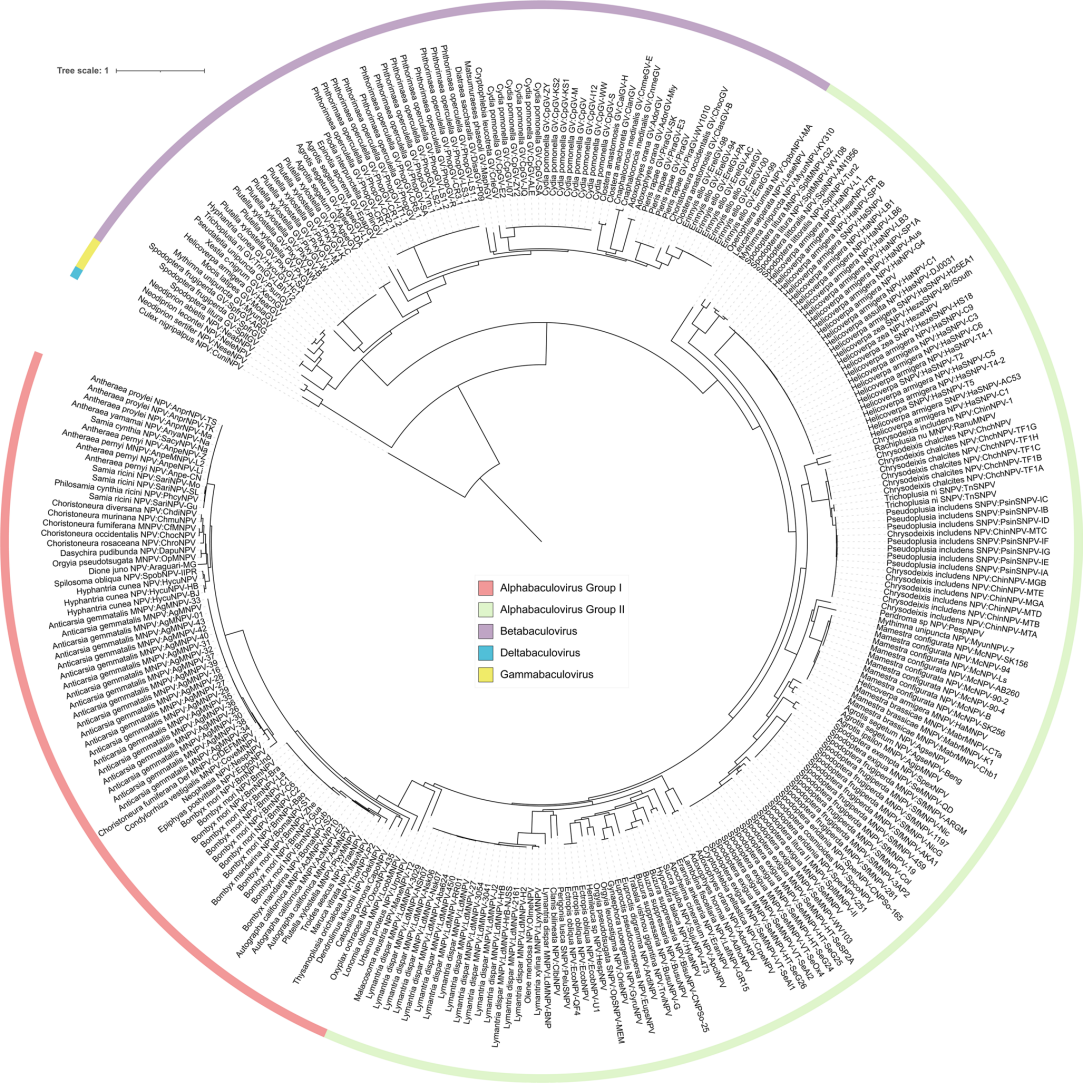
Maximum likelihood phylogeny of the family baculoviridae utilising the virus_odb10 (*n* = 12 orthologs) in Buscogeny

To assess Buscogeny’s utility for viral genomes we applied it to a dataset of baculovirus genomes corresponding to the same accessions used inCerrudo et al. (2023) study of the family (Cerrudo 2023). Using the virus_odb10 lineage dataset, Buscogeny identified the conserved single-copy orthologs and produced a concatenated alignment for phylogenetic inference. The resulting maximum likelihood phylogeny recovered the four major baculovirus genera, with alphabaculoviruses further resolved into groups I and II, consistent with previously reported results (Fig.8).

This demonstrates that Buscogeny can capture the expected evolutionary structure of baculoviruses using an orthology-based approach, without requiring a priori knowledge of the core protein set.

Although the exact core gene set differs from the canonical 38 proteins traditionally used (virus_odb10, orthologs *n* = 12), the Buscogeny phylogeny recapitulates the main clades and reflects the accepted taxonomy of the family using less genes. This highlights the potential of Buscogeny to facilitate viral phylogenomic inference in groups such as large dsDNA viruses, where conserved orthologs are present. Importantly, this analysis demonstrates that Buscogeny is not restricted to large, gene-rich eukaryotic genomes, but can also provide informative phylogenies for more compact viral genomes. Nevertheless, we note that the approach may be less applicable to viruses with very small genomes or limited sets of shared genes, where traditional annotation-dependent or alignment-based workflows may remain necessary.

## Discussion

Generating a species tree for fungi utilising whole genomes presents several challenges. Multilocus Sequence Alignments (MLSA), are a popular approach for phylogenetic studies, which requires careful selection of gene markers that are both conserved and variable enough to reflect evolutionary distances between species (Glaeser and Kämpfer 2015). In fungi, this selection is complicated by the high genetic variability and often sparse genomic data available for many species. Identifying genes that consistently correlate with species delineation involves extensive comparative genomic analysis, which can be hampered by incomplete or poorly annotated genomes. Unlike bacterial genome annotation, annotating eukaryotic genomes is not straightforward. Eukaryotic gene prediction often relies on additional evidence, such as RNA-seq and proteome data, for ab-initio gene predictors to help identify species specific splicing of intro/exon boundaries (Haas 2011)and training of ab-initio models themselves (Hoff and Stanke 2019). As such, only a small proportion of fungal genomes contain annotations; as of September 2025, 6,313 of 23,360 (~ 27%) of fungal genomes on NCBI are associated with annotations.

Concatenated gene trees, which rely on aligning multiple genes across species, are often sensitive to missing data. In the case of genome assemblies, missing genes or partial gene sequences, particularly at contig ends, can lead to large gaps in concatenated alignments, which in turn may result in poorly resolved phylogenies. This issue is particularly pronounced when working with fungal genomes, as they are often less complete or well-annotated than bacterial genomes. To mitigate these challenges, Buscogeny offers users the flexibility to select ortholog datasets of varying resolutions, depending on the completeness and quality of the genome data available. For instance, Buscogeny can utilise larger, high-resolution OrthoDB datasets which are more comprehensive and robust to missing genes. On the other hand, for genomes with more gaps, Buscogeny also allows users to opt for smaller, more conserved orthologous datasets that focus on highly conserved genes. This choice can help ensure that a sufficient number of core genes are present across all species in the alignment, reducing the impact of missing or poorly annotated genes. Additionally, Buscogeny has the ability to produce alignments for only those isolates that meet a user-specified threshold of genome completeness, as well as generating visualisations for identifying poor genomes that may then be removed from analysis, further increasing the robustness of the final phylogeny by ensuring that data are not skewed by incomplete genomes.

In addition, Buscogeny leverages clipkit to reduce gapyness in the final alignment (Steenwyk 2020). Gap positions can negatively impact phylogenetic inference by reducing regions of the alignment that lack meaningful sequence variation. Removing all gap containing alignment positions in an alignment (strict-core) reduces informative sites drastically when compared to a soft-core approach (Taouk 2025). By default, Buscogeny applies a 5% gap content threshold, ensuring that alignments are not overly filtered, reducing the alignment of informative regions. A stricter core alignment (where gap-containing positions are removed) can drastically reduce the number of informative sites available for tree construction, which has been shown to lead to a loss of resolution.

Buscogeny has been previously used successfully for fungal phylogenetic inference, demonstrating its value in resolving complex evolutionary relationships and assessing population structure in challenging taxa, such as *Verticillium dahliae*(Webster 2022). More broadly, the general methodology of leveraging BUSCO-derived orthologs for phylogenetic inference is well-established (Steenwyk 2019; Hensen 2023; Pizarro 2018; Waterhouse 2018) and increasingly adopted in both prokaryotic and eukaryotic phylogenomics, encompassing bacteria, fungi, insects and higher level eukaryotes. Despite its growing popularity, this approach often requires a combination of manual steps, including quality control to investigate the presence/absence of orthologs, ortholog extraction, alignment, trimming, and tree inference, which can be time-consuming and relies on specific bioinformatic skills. Buscogeny addresses this gap by providing an easy-to-use, open-source command-line tool that automates these processes. Buscogeny also flexibly allows the use of different orthoDB datasets to assess a variety of species through multiple taxonomic levels, and in addition may be run to identify and account for bacterial recombination on a self-constructed, gene partitioned supermatrix. Through its integrated workflow, Buscogeny enables users to rapidly assess genome quality, extract orthologs, generate alignments, apply appropriate alignment filtering such as soft-core trimming and recombination filtering, and infer phylogenies, all with minimal user intervention. This streamlined and reproducible framework lowers the barrier to entry for researchers aiming to apply BUSCO-based phylogenetic approaches, making it particularly valuable for large-scale comparative studies and non-model organisms where annotation and data completeness may be limiting factors.

## Conclusion

The construction of robust species trees for eukaryotic organisms using whole-genome data is a complex task, particularly due to the inherent challenges in genomic variability, incomplete assemblies, and the difficulty of accurate gene annotation. In this study, we have demonstrated the utility of Buscogeny as an effective tool for generating high-resolution phylogenies by leveraging core orthologs, flexible dataset choices, and robust handling of recombination and alignment gaps.

## Supplementary Information

Below is the link to the electronic supplementary material.

Supplementary Material 1 (XLSX 108 KB)

## Data Availability

All genomes used in this study are readily available on GenBank and were accessed and downloaded using NCBI_genome_download, as specified in Sect. 3.1. Buscogeny is available for use under a GPL-3.0 license on GitHub.com.
